# China’s engagement with development assistance for health in Africa

**DOI:** 10.1186/s41256-017-0045-8

**Published:** 2017-08-09

**Authors:** Mohon Shajalal, Junfang Xu, Jun Jing, Madeleine King, Jie Zhang, Peicheng Wang, Jennifer Bouey, Feng Cheng

**Affiliations:** 1 Family Planning Association of Bangladesh (FPAB), Dhaka, 1000 Bangladesh; 2 Research Center for Public Health, School of Medicine, Tsinghua University, Beijing, 100084 China; 3 Department of Sociology, Tsinghua University, Beijing, 100084 China; 4 School of Public Policy and Management, Tsinghua University, Beijing, 100084 China; 5 School of Advanced International Studies, Johns Hopkins University, Washington DC, 20009 USA; 6 Department of International Health, School of Nursing & Health Studies, Georgetown University, Washington DC, 20037 USA

**Keywords:** China, Development assistance for health, Public health, Africa

## Abstract

**Background:**

As an emerging donor in health related development across the world, particularly towards Africa, the People’s Republic of China (PRC) has been increasing its influence within the field of global public health over the past few decades. Yet between the period of 2000 and 2013, little is known about the scope, scale and priority of China’s grant-making programs.

**Methods:**

Based on data sourced from the China Aid Database (version-1.2), descriptive analyses were applied to analyze the features of 531 health related projects that were undertaken between 2000 and 2013. Spearmen correlation was also performed to assess the relationship between China’s export and aid to recipient countries.

**Results:**

The total value of China’s grant-making programs in the health related sector between 2000 and 2013 was 5.67 billion USD, with 531 projects undertaken. During the five year period between 2004 and 2008, China had a contribution of 1.54 billion USD, which increased to 3.8 billion USD during the five year period between 2009 and 2013 – an 146.26% increase. In terms of specific diseases, China is most concerned with building an African public health system through donations targeted towards general health (313 projects), combating Malaria (115 projects) and maternal, neonatal and child health (MNCH), (12 projects). When it comes to recipient countries, if counted in total value, Zimbabwe received the most financial assistance from China, totaling 1.08 billion USD and 19 projects, while Angola and Tanzania received more projects - 30 and 29 projects respectively. In terms of the channeling of aid funding, most projects were targeted towards infrastructure, equipment and medicine (304 projects in total), followed by medical teams (189 projects). Moreover, there is a statistically significant relationship between aid to Africa and Chinese exports to Africa.

**Conclusion:**

During the past decade, Chinese aid projects played an important role in the African public health system through providing funding for infrastructure, equipment and medicine, training health professionals, as well as disease treatment. However, very limited attention was paid towards disease prevention, health promotion and awareness initiatives, and health education. Furthermore, serious questions were raised regarding the long-term financial sustainability and actual impact these projects have on health development.

## Background

As one of the largest emerging donors of foreign aid, China began to provide overseas assistance to other socialist countries shortly after its independence in 1949. Although China was still very poor, in 1950 it provided material assistance to Vietnam and North Korea. After the Bandung Asia-Africa Conference in Indonesia in 1955, China also began donating to other non-socialist developing countries [1]. Since that time, China has helped 40 African countries to develop their health systems by providing healthcare products and sending medical teams (roughly1000 medical staff), with an estimated cost of $60 million USD per year [2, 3]. Yet for China itself, significant disparities still remain in the overall health status between urban and rural citizens, and many Chinese citizens still lack access to high-quality medical services. Over the last decade China has gone from aid recipient to non-conventional donor in global development aid. While donations from developed countries to less developed countries have stagnated in recent years, aid flows from emerging economies such as Brazil, Russia, India, China and South Africa (BRICS) are playing an increasingly crucial role in global health development [4]. For example, China increased its contribution to global health assistance by $3 billion USD between 2000 and 2012 [5]. Meanwhile net official development assistance received by China decreased from 0.12% of China’s Gross National Input (GNI) in 2001 to 0.01% in 2010 [6].

China’s foreign aid policy exhibits distinct characteristics across different time periods - a product of its own development experience and of the needs of aid recipient countries. Since the guiding principles of China’s foreign aid, known as the Eight Principles for Economic Aid and Technical Assistance to Other Countries, was put forward in the 1960s China has been constantly improving and developing in the sphere of foreign aid. China’s foreign aid falls into the category of South-South cooperation, which concerns the notion of mutual help between developing countries. On the 10th of July, 2014, the Chinese State Council published a White Paper on China’s foreign aid (2014), which stipulates the policy of mutual benefit, and respect with no conditionality attached [7].

Recipient countries sometimes request for China to provide them with aid focusing on the health sector. For example, many developing countries hope that China can assist in helping to build local capacity of public health delivery system through human resources training, building large public health infrastructure, as well as the development of natural medicine and other medicinal industries [8]. In addition, there is a strong desire for assistance with helping establish a sound medical and public health service system, for example focusing on help with technology for dealing with tumors, cardiovascular related issues, the trauma department in orthopedics, medicine and treatment for rehabilitation and other such fields [9]. Furthermore, developing countries hope that health aid from other countries can help realize the localization of production and supply of the pharmaceutical industry [10, 11].

In 2014, the Chinese government published a White Paper on Aid in 2014 and the Ministry of Commerce reported in its official yearbooks a list of comprehensive projects completed in recipient countries between 1990 and 2005 [12]. However, no detailed information is available on the different types of development assistance and the correlations between exports from China and aid from China. Although a previous study based on China Aid database version 1.0, estimated that the largest share of money went to the health sector during the period 2000 to 2011 ($696 million USD for 154 projects) [12]. Using updated database version 1.1, Grepin et al. analyzed 255 projects in the Health and Water and Sanitation sector during 2000 and 2012, and estimated that the monetary value of Chinese health development assistance was about $3 billion USD [5]. However, an updated version of China Aid Data with information about more projects is now available. Therefore, with the new version of database 1.2, the purpose of this study was to provide an updated analysis on the scope, scale and priority of public health projects received by African countries from China. Moreover, based on available data, the correlation between export from China and aid from China was also examined.

## Methods

### Data sources

The China Aid Data Database (version 1.2) was used for our analysis. The database tracks development projects (a total value of $40 trillion USD) funded by different donors around the world [13], including development projects (2648 projects) funded by the Chinese government during the period 2000 and 2013 [14]. Significant improvements have been made to create Database version 1.2 particularly through the refining of data collection methodology to identify new sources of primary data and detecting systematic sources of bias that can be corrected or minimized. In an attempt to track unreported financial data from different nonconventional donors such as China, BRICS governments and Gulf states, Aid Data developed a media based open-source data collection methodology known as “Tracking Under-Reported Financial Flows (TUFF)”, which is employed to collect primary project-level data from different media. TUFF methodology collects project information from different data sources such as media reports, aid information management systems, scholarly articles and government and embassy recipient country websites. The following procedures were carried out to improve the quality of data: (a) Gathering data through Factiva, which draws together roughly 28,000 media sources in 23 languages; (b) Gathering together information from government and non-government reports, research, and case studies; (c) Investigating research information used to verify and incorporate missing information about aid projects. The data on Chinese exports for the period of 2000 to 2013 was obtained from the World Bank website.

### Statistical analysis

Projects were categorized in line with the creditors reporting sectors (CRS) coding system and by the type of projects: Health-120, Population Policies/Programmes and Reproductive Health-130 and Water Supply and Sanitation-140 were categorized as public health interventions (Table 1 shows the standard of categories on projects). Projects were also categorized according to the state of progress, such as: being implemented, completed and pipeline commitment, cancelled, pipeline-pledge, and suspended. Pipeline commitment refers to a verbal commitment without any written form of commitment, whilst pipeline pledge projects have a certain degree of written commitment. Only projects that are completed, being implemented and pipeline pledge projects were included in the analysis. The data was also categorized according to the form of measuring Official Development Assistance (ODA), such as: other official flows (OOF) including military, vague, aid from Non-Governmental Organizations, official investment, commercial activities and foreign direct investment. Only projects that are similar to ODA Like, OOF Like and Vague (either ODA Like or OOF Like) were included in our analysis. A key word search was performed to identify health related projects in other sectors. The following is the Example of key words performed to identify related projects: (China, Chinese or Chin*) AND (Angola, Angolan or Angol* or Luanda) AND (assistance, grant, loan, concession*, donat*, donor, interest-free, interest, preferential, joint fund, finance, package or aid).

**Table 1:** The standard of categories of projects

Standard of categorization	Categories
Projects according to CRS coding system	Health
	Population Policies / Programmes and Reproductive Health
	Water Supply and Sanitation
	Government and Civil Society
	Emergency Response
	Support to Non-governmental Organizations (NGOs) and Government Organizations
	Other Multi-sector
Health Aid Projects by Disease	General Health
	Cholera
	Ebola
	Emergency
	Eye
	HIV/AIDS
	Influenza
	Lasa Fever
	Malaria
	Renal
	Sexual and Reproductive Health (SRHR)
	Tuberculosis
Health Aid by Activities	Awareness raising
	Education
	Infrastructure, equipment and medicine
	Medical Team
	Research
	Scholarship
	Training
	Others

Descriptive statistics methods were used to analyze the features of aid projects. Spearmen correlation was performed to test the relationship between Chinese aid and exports. IBM SPSS 24.0 software was used for the statistical analyses, with an alpha level of *p* < 0.01.

## Results

### Number of projects and Total value of donations by different sectors

**Fig. 1:**
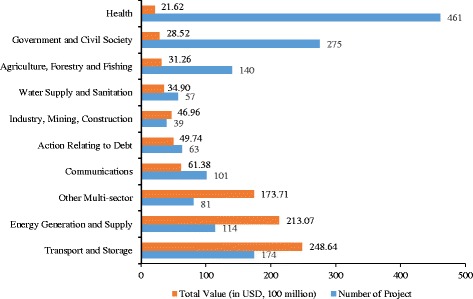
Number and total value of grants distributed by the Chinese government among different sectors (Top 10 sectors, excluding unallocated/unspecified sectors)

During the period between 2000 and 2013, China donated $104.35 billion USD into 2286 development projects across 24 different sectors. The transportation and storage sector received the largest amount of money, with $24,863.78 million USD for 174 projects. In terms of the amount of donations, the health sector stands at number 10, with $2162.338 million USD in donations. However, the health sector ranked first in terms of number of projects, with 461 projects out of the 2286 projects in total (Fig. 1).

### Projects in the health related sectors

In the health related sectors, most of the projects (461) were in health, while the water supply and sanitation sector received the largest amount of funds ($3489.532 million USD), with 57 projects (Table 2). The sector of government and civil society received 2 projects and $15.59524 million USD, population policies/programmes and reproductive health received $1.461589 million USD with 8 projects. Moreover, the emergency response sector received 1 project ($0.032633 million USD), and the NGO sector and the multi-sector both received 1 project each.

**Table 2:** Total number and value of projects in the public health sector

Sector	Number of projects	Total value (in million, USD)
Health	461	2162.338
Population Policies / Programmes and Reproductive Health	8	1.461589
Water Supply and Sanitation	57	3489.532
Government and Civil Society	2	15.59524
Emergency Response	1	0.032633
Support to Non-governmental Organizations (NGOs) and Government Organizations	1	-
Other Multi-sector	1	-
Total	531	5668.96

### Number and Total value of public health projects by year

**Fig. 2:**
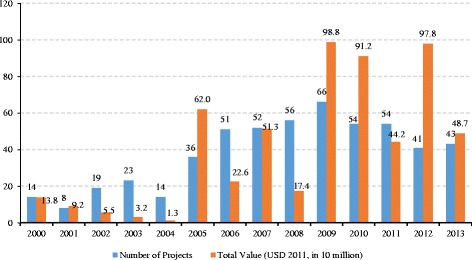
Number and total value of public health projects between 2000 and 2013

According to Fig. 2, only 14 projects valued at a total of $137.80 million USD were undertaken in the year 2000. After a continuous decline in terms of aid value up until the year 2004, in 2005 the number of projects and the amount of aid increased sharply to 36 and $619.59 USD million respectively, with both reaching their peak in 2009 (66 projects and $987.74 million USD). The amount of aid also more than doubled, increasing from $1.54 billion USD between 2004 and 2008, to $3.8 billion USD between 2009 and 2013.

### Geographical distribution of public health projects

Table 3 shows that Zimbabwe, which received 19.08% of China’s foreign aid and $1081.83 million USD between 2000 and 2013, is the largest recipient country among all, and that Angola, Cameroon and Ghana also received a significant portion with $1029.11, $957.10, and $841.70 million USD respectively. The number of projects and total value received by other countries are shown in Table 3. An interesting point to note is that the amount of aid received by these four countries mentioned above (Zimbabwe, Angola, Cameroon and Ghana) amounted to 68.96% of total development assistance for health distributed by the Chinese government.

**Table 3:** Top 10 recipient countries by cumulative total of grants awarded by the Chinese government

NO.	Country	Total value (USD 2011, in million)	No. of projects	% of total grants money received	% of the projects having information on monetary value
1	Zimbabwe	1081.83	19	19.08	42.01
2	Angola	1029.11	30	18.15	83.33
3	Cameroon	957.10	19	16.88	47.36
4	Ghana	841.70	17	14.85	52.94
5	Cote D’Ivoire	254.01	6	4.48	100.00
6	Sudan	149.24	15	2.63	40.00
7	Kenya	131.55	15	2.32	66.67
8	Mauritius	116.13	7	2.05	71.42
9	Uganda	108.76	24	1.92	54.16
10	Zambia	73.68	19	1.3	26.31
Total	-	4743.11	171	83.66	-

### Development assistance for health by disease

**Fig. 3:**
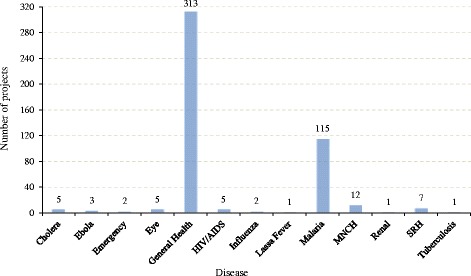
Number of projects by disease (WASH not included)

A total of 313 projects were related to the general health sector ($1819.27 million USD), which includes projects targeted at building comprehensive health facilities, such as pathology, lab, surgery and health ministry facilities, as well as projects targeted towards general health. Compared to other diseases, addressing Malaria seems to be the highest priority of Chinese government, with Malaria related projects constituting 115 projects out of 161 disease specific projects ($179.69 million USD). Moreover, maternal and child health (MNCH) follows malaria, ranking as third in terms of project numbers, and receiving 12 projects and $145.47 million USD (Fig. 3). Sexual and reproductive health (SRH) received 7 projects while HIV/AIDS, eye health, and cholera all received 5 projects each. Furthermore, the Chinese government initiated projects to respond to the outbreak of Ebola (3 projects), Influenza (2 projects) and Lasa Fever (1 project).

### Development assistance for health by activities

**Fig. 4:**
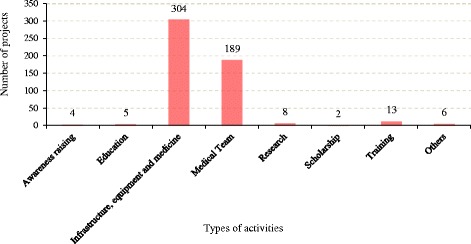
Distribution of Chinese public health assistance by types of activities during 2000–2013

A total of 304 projects ($4427.08 million USD) in aid were targeted towards infrastructure, equipment and medicine (Fig. 4). The second largest number of projects went to sending medical teams to different countries, with 189 projects (totaling $65.20 million USD). Research activities received the third largest amount of aid, with 8 projects ($1018.74 million). Scholarships, training, education, and health awareness raising initiatives received 2, 13, 5, and 4 projects respectively. In addition, the 20 largest projects received 73.73% of the total health related sector money. Most of the projects were implemented by government agencies (75.60%), while NGOs, foundations, private sector and public-private organization were involved in execution of 24.39% projects.

### Correlation between Chinese exports and aid

The results of Spearmen correlation analysis showed that there is a statistically significant association between Chinese development assistance for health and exports from China (*P* = 0.004) (Table 4). However, there is no statistically significant association between Chinese exports and specific types of health aid, including General Health (*P* = 0.152), Population Policies/ Programmes and Reproductive Health (*P* = 0.538) and Water Supply and Sanitation (*P* = 0.163). Moreover, there is no statistical significance between the number of health aid projects and China’s exports (*P* = 0.400).

**Table 4:** The correlation between Chinese exports and specific types of health aid

Independent variables	β	*P* value
General Health	0.217	0.152
Population Policies/Programmes and Reproductive Health	0.319	0.538
Water Supply and Sanitation	0.288	0.163
Total aid	0.416	0.004
Number of projects	−0.127	0.400

## Discussion

China provides aid funds in the form of development assistance for health to 48 out of the 54 African countries (the 6 countries not receiving aid being Burkina Faso, Ivory Coast, Sao Tome and Principe, Swaziland, Gambia, and Egypt). Out of these six countries, Burkina Faso, Sao Tome and Principe, and Swaziland currently hold diplomatic relations with Taiwan [15] and Gambia held diplomatic relations with Taiwan until 2013 [16, 17]. However, although not in the sector of public health, Egypt and Sao Tome and Principe received aid projects from China in other sectors (such as in industry, mining, construction, education, energy, trade and tourism), receiving 50 and 4 projects respectively.

The top 10 countries received 83.66% of the total projects provided by China, and four countries: Zimbabwe, Angola, Cameroon and Ghana, received around 69% of the total funding, which raises questions as to why some African countries enjoy a comparatively privileged recipient status compared to other African countries. Although Grepin et el. found that natural resources were not associated with the nature of China’s development assistance in health, Africa is the second largest source of crude oil for China, while Angola is the one of the largest suppliers of oil. With a ‘no strings attached’ policy, China is providing soft loans to mineral-rich fragile African countries, building their infrastructure and supporting such countries against international pressure for domestic reforms [18–22]. However, Chinese aid preferences can not be described only with a single variable. Therefore, further analysis and research using multiple variables is necessary.

Although China is more interested in building up an overall health system in recipient countries compared to disease-specific focused projects, aid projects related to Ebola, Influenza and Lasa Fever also serve as evidence of China’s engagement in the African epidemiological crisis. Among all diseases, Malaria received the highest priority in Chinese health development assistance to Africa. In 1993, WHO approved Chinese traditional medicine for treatment of malaria and since 1996, China made the use of Chinese malaria medicine mandatory in health aid projects. There has long been a high need for overseas assistance to combat malaria in African countries. China also had its own serious problem with malaria which led Chairman Mao Zedong in 1967 to set up a research team to find a cure for malaria [22, 23].

The way that China actually delivers its overseas assistance projects is an interesting point of consideration. In most cases, projects are implemented by either state-owned enterprises or Chinese private companies. In some cases, projects are implemented by the Government of the recipient country. The engagements of NGOs were also observed, but in only two projects: one in a regional eye-care project, and another in an infant malaria project in Ghana. These findings suggest that China is a not a passive donor, and actively engages in the implementation of projects. This is beneficial for rapid implementation, and increases the cost-efficiency of projects, as it is very difficult to sustain and carry projects forward by weak health system and management in African countries [24]. Involving local people may also bring positive effects in the long term by creating a sense of local ownership, as aid can deliver a more sustained result when local beneficiaries are involved and have ownership over the project. For example, China has actively carried out the so-called “Brightness Action” to help treat more eye diseases and since 2003, China has sent medical teams to North Korea, Cambodia, Bangladesh, Vietnam, Pakistan and other Asian countries to treat local patients who have eye diseases. In addition, doctors from the China Medical Team have collaborated with and trained local doctors whilst treating the patients in Africa [25]. Furthermore, local medical personnel were actively engaged and received capacity building support during these projects. China is also encouraging African foreigners to actively take part in aid projects. For example, foreigners especially Africans can apply for aid projects from China and carry them out by themselves [26].

Aid funds from China are found in three different forms: free aid, interest-free loans and concessional loans [25, 27]. Free aid focuses on helping recipient countries to build small and medium sized construction projects and implement human resource development, technical cooperation, material assistance and emergency humanitarian assistance. Interest-free loans are mainly used to help recipient countries build public facilities and livelihood projects. Concessionary loans are mainly used to help recipient countries to build production projects that will promote economic and social benefits, large and medium sized infrastructure projects, or to provide larger equipment, mechanical and electrical products. Direct health aid in terms of equipment may be being counted as a form of export from China to recipient countries. So there may be a direct relation between health aid and exports. Indeed, spearmen correlation performed in the study showed a statistically significant association between Chinese aid and exports (*P* = 0.004), although there is no statistically significant association between Chinese exports and specific types of health aid (*P* > 0.05). Moreover, unlike other traditional donors, China provides aid to fragile African states without attaching any political reform conditions. However, the basis of cooperation is to promote mutual development, including promoting greater exports to recipient countries to meet the production needs of recipient countries and promote economic development within recipient countries. On the other hand, China can also expand imports of raw materials from recipient countries. With the continuous development of China’s economy, the demand for natural resources to fuel the economic boom is increasing. Therefore, recipient countries can take advantage of the demand for rich raw materials to greater develop their local industries and further cooperate with developed countries [28]. Furthermore, with Western countries recently withdrawing or reducing their developmental assistance and engagement with African countries, China has come forward with an overall assistance package in the form of monetary funding, technical expertise, and international influence [29] - which has also facilitated the export of cheap and affordable products from China to Africa. Finally, as an important form of human capital, health is the key factor of individual, social and national productivity, and has a vital role in promoting the growth of national income. Good health increases human capital and productivity and promotes the growth of personal income, which helps improve household consumption and domestic demand overall [30–32]. At the same time, according to Keynes’s theory of consumption (the key points within the absolute income hypothesis), actual consumer spending is the steady function of actual income, and the income level of residents has a direct linear relationship with the level of consumption – which is a positive relationship. Thus improving health will promote the level of consumption [33], which also demonstrated that health aid to recipient countries may promote local consumption and exports from China to African countries.

In addition, within these programs, health awareness and health education programs for local people is severely lacking, with only four projects addressing the raising of health awareness in some form. With proper implementation, health promotion and disease prevention - which are more cost-effective than disease treatment, will have a greater impact on health development overall. Furthermore, without health promotion, the demand for health services may not increase significantly, even if healthcare services are available. With universal health insurance coverage reaching 95% in less than a decade, China itself has made significant achievements in raising health awareness through the launching of very successful public health campaigns and the introducing of an effective health financing system [34].

In our study, most of the projects (304 projects worth $4427.08 million USD in funding) were targeted towards infrastructure, equipment and medicine. Meanwhile, Western countries are not responding to African countries’ needs for infrastructure development [35]. In a study on aid and humanitarianism, Ward Warmerdam (2013) stated that China feels that infrastructure (for example building roads) is essential for development, which requires huge investments [36]. However, many Western donors do not as much appreciate that infrastructure is an important sector requiring investment [35]. Yet, with China having its own various issues regarding development, its capacity to provide health development assistance may currently be somewhat limited. Moreover, most of African countries do not have the sufficient financial capacity to sustain and maintain facilities built through project-based initiatives. In Africa overall, like the health service delivery system, the health financing system is also weak [37]. From our analysis, we could not find any project that dealt with the issue of health financing. Therefore, it is necessary for both China and its partners in Africa to work together to maximize the benefits for African citizens in this regard.

It is very important to point out that China is considering deepening its experience in delivering aid projects. Beijing has already begun this process by working with the United Sates of America in supporting the African Union to develop the African Centre for Disease Control and Prevention (ACDCP) [38]. Furthermore, China also initiated the Global Health Support Program (GHSP), a £12 million pound China-UK partnership project (2012–2017) which aims to increase and improve China’s contribution to disease control and maternal health towards low and middle income countries by spreading the Chinese experience in improving health outcomes and strengthening health systems [39]. Although it is clear that Chinese assistance has helped African countries to develop their health systems, our results raised three serious questions about the nature of Chinese health development assistance: (i) Should China only focus on hospital-based curative services when promoting health education, and might education focusing on behavioral change be of greater benefit to people living in poverty? (ii) Can aid foster development if the financial aspect of projects and the importance of institutional and policy reform of the recipient countries are ignored? (iii) Should we rely solely on government agencies in delivering aid but forget or underestimate the role that the private sector can provide in promoting health development?

### Policy implications

China should consider (a) Investing more in educating people about disease prevention and treatment, with a focus in primary prevention and treatment. Mass education and awareness campaigns should be designed based on research and evidence about various behaviors and misconceptions held by citizens, local culture and other socio-economic and geographic factors (b) Aid can only deliver a sustained result when local beneficiaries are involved. Therefore in addition to involving government officials, it is crucial to consult and involve local people in the designing and implementation of projects. (c) China is supporting African countries in building a large amount of health facilities. However, without a proper health financing system the sustainability of the projects is not certain. Following its own experience, China may like to consider supporting African countries to develop their own health financing or health insurance systems in the future.

### Limitations

Some limitations exist in the study due to the unavailability of data, including for the following reasons. Firstly, among the 531 projects included in the analysis, only 206 projects have a monetary value assigned. Secondly, the Chinese government began providing donations to multi-lateral organizations such as the World Health Organization (WHO) and the Global Alliance for Vaccines and Immunizations (GAVI), however, yet data for these projects is currently not available. Thirdly, significant amount of assistance was directed towards infrastructure development. However, it is impossible to further analyze and provide information on the resources devoted to the establishment of infrastructure at primary care level and tertiary hospital because of the limited data. It was also very challenging to categorize projects, as some projects can be placed into more than one category. For example, sending medical teams can also be categorized as training.

## Conclusion

China is playing an increasingly important role in the African public health sector. However, only a handful of countries have received priority in receiving Chinese development assistance for health. The findings from our study confirm that: China is engaged in helping African countries to develop their health systems by helping develop infrastructure and supplying equipment and medicine, as well as training health professionals and funding disease treatment. However, very limited attention has been paid to addressing disease prevention, health education, and health promotion and awareness. Furthermore, financial sustainability and the involvement of NGOs and the private sector has been relatively ignored, which raises questions regarding the long-term sustainability and impact that Chinese health aid projects can have in Africa.

## Abbreviations

ACDCP: African Centre for Disease Control and Prevention
BRICS: Brazil, Russia, India, China and South Africa
CRS: Creditors reporting sectors
GAVI: Global Alliance for Vaccines and Immunizations
GHSP: Global Health Support Program
GNI: Gross National Input
MNCH: Maternal, Neonatal and Child Health
NGO: Non-Governmental Organization
ODA: Official Development Assistance
OOF: Other official flows
PRC: People’s Republic of China
SRH: Sexual and Reproductive Health
TUFF: Tracking Under-Reported Financial Flows
UK: United Kingdom
USA: United States of America
WHO: World Health Organization
